# System-based insights into parasitological and clinical treatment failure in Chagas disease

**DOI:** 10.1128/msystems.00038-24

**Published:** 2025-01-07

**Authors:** Luis Ernst, Giovana C. Macedo, Laura-Isobel McCall

**Affiliations:** 1 Department of Chemistry and Biochemistry, San Diego State University, San Diego, California, USA; University of California San Diego, La Jolla, California, USA

**Keywords:** drug development, antiparasitic treatment, treatment failure, Chagas disease, *Trypanosoma cruzi*, drug resistance, pathogen persistence, metabolomics, genomics

## Abstract

Infectious disease treatment success requires symptom resolution (clinical treatment success), which often but not always involves pathogen clearance. Both of these treatment goals face disease-specific and general challenges. In this review, we summarize the current state of knowledge in mechanisms of clinical and parasitological treatment failure in the context of Chagas disease, a neglected tropical disease causing cardiac and gastrointestinal symptoms. Parasite drug resistance and persistence, drug pharmacokinetics and dynamics, as well as persistently altered host immune responses and tissue damage are the most common reasons for Chagas disease treatment failure. We discuss the therapeutics that failed before regulatory approval, limitations of current therapeutic options and new treatment strategies to overcome persistent parasites, inflammatory responses, and metabolic alterations. Large-scale omics analyses were critical in generating these insights and will continue to play a prominent role in addressing the challenges still facing Chagas disease drug treatment.

## INTRODUCTION

Chagas disease (CD) is a neglected tropical disease caused by the protozoan parasite, *Trypanosoma cruzi* (*T. cruzi*) (1). Mammalian infection is by contact with infected blood-sucking triatomines, contaminated food, maternal-to-fetal transmission, and, increasingly rarely, blood transfusions with contaminated blood or tissue transplants (2–4). *T. cruzi* epimastigotes replicate in triatomines and differentiate into metacyclic forms in their hindgut. Upon transmission to mammals, trypomastigotes invade mammalian cells. There, they differentiate to proliferative amastigotes. Amastigotes then differentiate back into trypomastigotes (5).

CD proceeds through acute, indeterminate, and chronic stages. In the acute stage, nonspecific symptoms can occur soon after infection. The indeterminate phase is asymptomatic and can last 10 to 30 years. When untreated, ~30% of infected people develop a symptomatic chronic phase with cardiomyopathy, digestive megasyndromes, or both (6, 7).

*T. cruzi* initiates and sustains the mechanisms associated with tissue damage in CD (8). Thus, modern CD drug development has focused on drugs that kill *T. cruzi* (*9*). There are two antiparasitics in clinical usage worldwide, benznidazole (Bz) and nifurtimox (NFX), which nevertheless fail to clear *T. cruzi* or improve CD symptoms in some patient groups (10–12). The inability of a treatment regimen to clear all *T. cruzi* is defined as a parasitological treatment failure, which may or may not be accompanied with symptomatic disease. In parallel, even patients who successfully achieve parasitological clearance may continue to present symptoms or even die from cardiac CD (10), which is defined as a clinical treatment failure.

In this review, we cover the current knowledge on parasitological and clinical treatment failure in the context of CD and highlight gaps in the field, with emphasis on the roles of omics technologies. Resources for clinical trials in CD are limited; therefore, a deep understanding of parasitological and clinical treatment failure is crucial to reduce drug development costs and increase the likelihood of success (13).

## MECHANISMS OF PARASITOLOGICAL TREATMENT FAILURE

**Fig 1:**
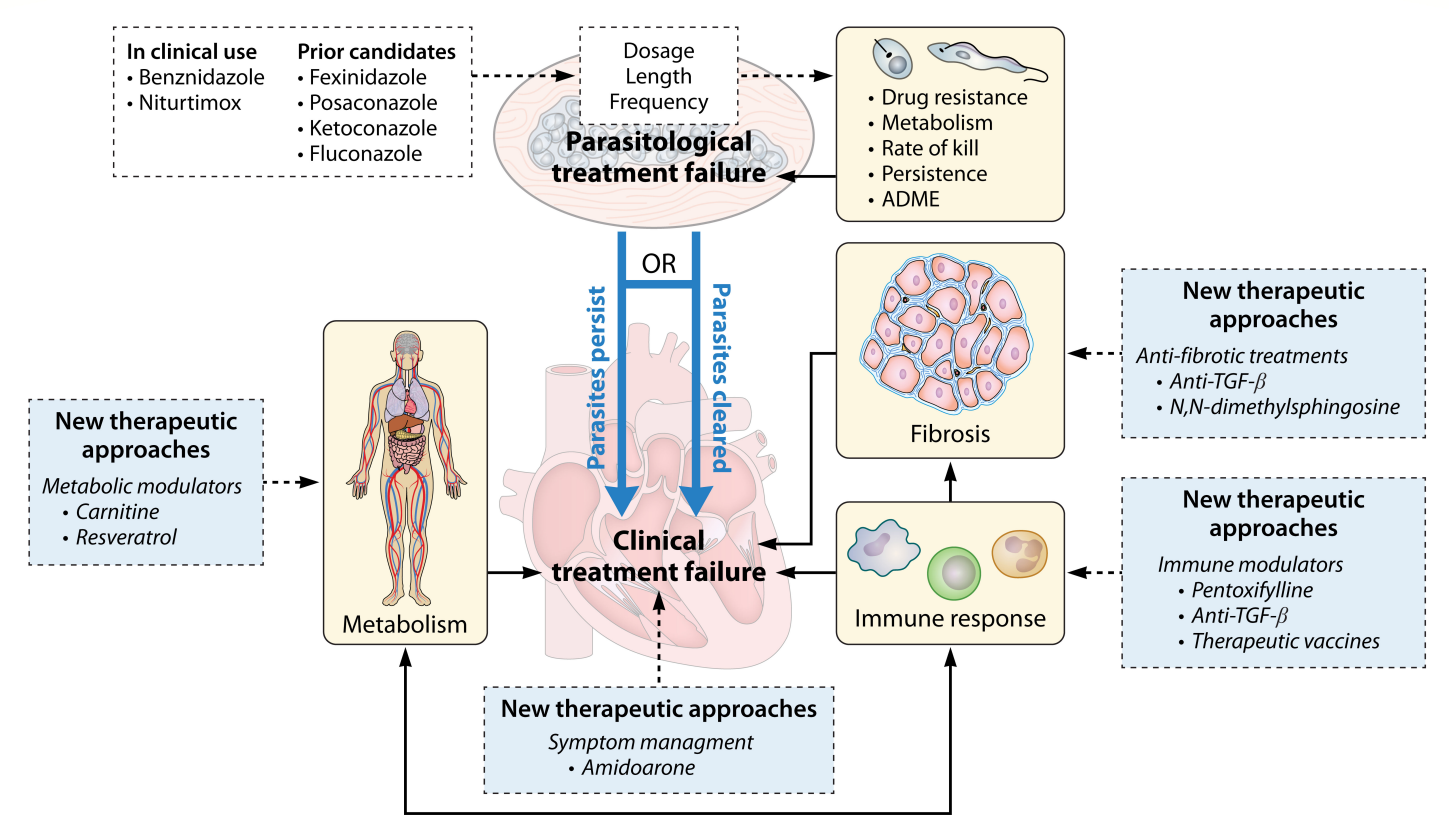
Causes of parasitological and clinical treatment failure and examples of current approaches to mitigate them. Clinical treatment failure can be observed with or without parasite clearance (blue arrows). ADME, absorption, distribution, metabolism, and excretion. Arrows show the direction of impact. Dashed lines represent new treatment approaches.

Parasitological treatment failure is indicated by the inability of treatment to kill *T. cruzi*. Factors contributing to parasitological treatment failure are persistent intracellular non-dividing amastigotes, resistant parasites, drug metabolism, and low drug concentrations at the site of parasite localization (Fig. 1) (14–20).

### Parasite replication rates and persistence

**Fig 2:**
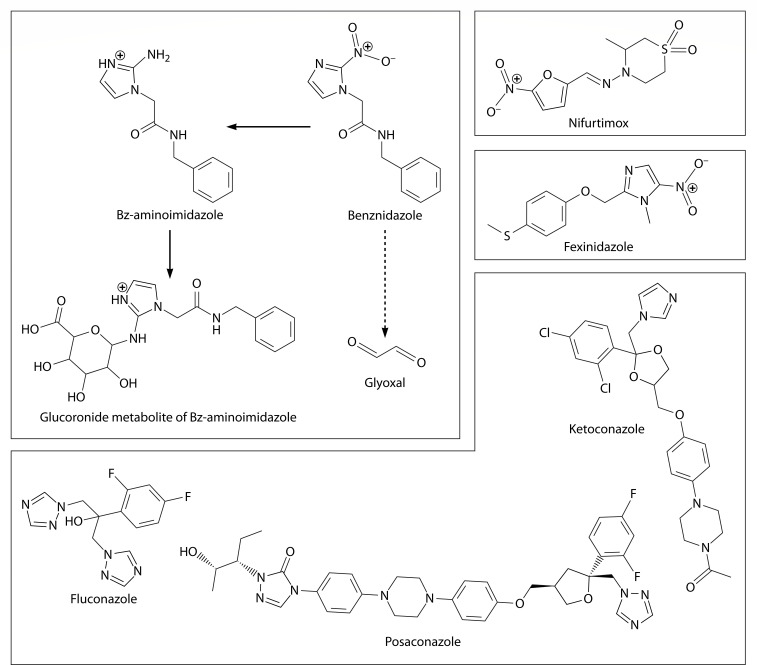
CD treatments in clinical usage worldwide, selected experimental treatments discussed in the text, and selected Bz metabolites.

Lanosterol 14alpha-demethylase (CYP51) inhibitors, including posaconazole (Fig. 2) and fosravuconazole, were unable to induce parasitological cure in humans (21–23). Mechanism of action involves inhibition of parasite membrane sterol production, so slower-growing strains are more resistant (24). Non-proliferating intracellular amastigotes survive Bz and possibly posaconazole treatment and can re-initiate replication and differentiation days or weeks after drug removal *in vitro* and *in vivo*. Dormancy classification in the context of *T. cruzi* lacks uniformity. However, most authors classify dormant amastigotes as amastigotes that did not replicate for at least 72 h after infection but are able to re-initiate replication (20, 25, 26). Dormant amastigotes appear spontaneously after host cell infection *in vitro* (20), but DNA damage increases the number of dormant amastigotes. Homologous recombination appears to play a role in dormancy signaling, as reduced levels of TcRAD51, a protein involved in repairing DNA double-stranded breaks, results in fewer dormant amastigotes *in vitro* (26).

Information is lacking as to whether the dormant amastigotes are in G0 or G1 of the cell cycle. However, a temporary pause in G1 is more likely than G0 because dormant amastigotes can reinitiate replication following drug removal *in vitro* (25). Non-replicating amastigotes can, however, transform into trypomastigotes, which are invasive and subsequently transform into dormant and replicative amastigotes, supporting the idea that dormant amastigotes are only cell cycle arrested. Reinitiation of replication was also shown *in vivo* (20). Dumoulin et al. identified a greater number of amastigotes in the G1 stage in response to 24 h of Bz exposure or nutrient restriction *in vitro* and suggested that increased doubling times rather than complete growth arrest were the cause (27). However, environmental stressors, such as Bz or nutrient restriction, cannot alone explain the grow arrest because dividing and non-dividing amastigotes co-exist in the same cell *in vitro* and *in vivo* (20, 28).

### Resistance

Strain-dependent resistance to Bz and NFX occurs naturally in *T. cruzi*. Lower parasitological clearance after treatment with Bz in Colombia and El Salvador compared to Brazil suggests that natural resistance may play a role in parasitological treatment failure in the clinic (10, 29). There is some evidence that this could be due to differences in natural drug susceptibility between TcI and TcII *T. cruzi* strains (29). Likewise, strains with a range of *in vitro* Bz susceptibility have been isolated from patients (30, 31). For example, the drug transporter ABCG1 is upregulated in Bz-resistant strains compared to Bz-susceptible strains *in vitro* (32), although no association between *TcABCG1* single-nucleotide polymorphisms (SNPs) and drug resistance was observed (33).

Higher IC_50_ was observed in three post-treatment isolates compared to three strains isolated pre-treatment from different patients (31). However, these studies did not perform temporal sampling from the same patient. Some but not all comparisons of *T. cruzi* genetic diversity within a given patient pre- vs post-treatment identified genetic signatures suggestive of drug selection, albeit confounded by the possibility of differential parasite dynamics between tissues and blood over time (34, 35). In contrast, the *in vitro* Bz susceptibility analysis of epimastigotes isolated from chronic CD patients pre-treatment could not predict treatment outcomes (36). The epimastigote form is not clinically relevant but presents a similar Bz susceptibility to amastigotes (29).

In response to these limitations and the challenges of isolating parasites from patients post-treatment, the current literature on Bz and NFX resistance mechanisms is predominantly based on *in vitro* selection studies. Transcriptome differences were observed by RNA-seq between Bz-resistant strains obtained after *in vitro* drug pressure and Bz-sensitive strains, including genes involved in metabolic pathways (16). RNA sequencing, chromosomal analysis, and quantitative reverse transcription PCR demonstrated that *in vitro*-induced Bz and NFX resistance is associated with mutations of the trypanosomal nitroreductase (TcNTR), decreased TcNTR gene expression, or differences in the TcNTR copy number, with cross-resistance to other nitroheterocyclic drugs and to compounds from other chemical classes, such as posaconazole (14, 16, 37–39). The loss of NTR function in the bloodstream form of the parasite *Trypanosoma brucei* is also correlated with resistance to Bz and NFX, as revealed by RNAi screens (40). TcNTR is essential for the activation of the pro-drugs Bz and NFX into their active metabolites (41), which bind and damage parasite proteins, nucleic acids, and lipids (42). MS-based metabolomics revealed a broad range of Bz biotransformation products in *T. cruzi* epimastigotes, such as a dihydroxy-dihydroimidazole-derivative, 2-(2-amino-1H-imidazol-1-yl)-N-benzylacetamide, and Bz-glutathione adducts *in vitro* (17). Glyoxal and N-benzyl-2-guanidinoacetamide are the reported biotransformation products of a dihydrodiol Bz derivative. However, only N-benzyl-2-guanidinoacetamide was detected by reversed-phase ultra-high performance liquid chromatography–quadrupole time of flight mass spectrometry (UHPLC–QTOF-MS) and UHPLC–Q–linear ion trap–MS of human urine samples (43).

Additional mechanisms of resistance identified through *in vitro* Bz selection and *in vitro* experimentation include detoxification of Bz by the *T. cruzi* cytochrome P450 reductase B (TcCPR-B) (44), neutralization of active drug metabolites that are substrates of the *T. cruzi* aldo-keto reductase (TcAKR), as revealed using UV spectroscopy (45), neutralization of free radicals or electrophilic metabolites produced after Bz transformation by the antioxidants trypanothione and glutathione and enzymes, such as ascorbate peroxidase (16, 46, 47), or overexpression of ABC transporters, such as TcABCG1 (48, 49).

Variations in CYP51 inhibitor sensitivity between *T. cruzi* strains have been postulated to be caused by different CYP51 sequences or expression levels (50, 51). Indeed, treatment with posaconazole and fluconazole increased the CYP51 protein levels (51).

However, *in vitro* findings from drug pressure-selected *T. cruzi* do not always translate to naturally occurring Bz resistance, as demonstrated by studies showing gene expression changes in iron-containing superoxide dismutase-A (TcFeSOD-A) or cytosolic and mitochondrial tryparedoxin peroxidases after drug selection *in vitro*. However, no significant differences were found between naturally Bz-susceptible and -resistant strains (52, 53). Likewise, different SNPs were observed in TcNTR in *in vitro* selected strains vs clinical isolates, and there was no clear association between TcNTR haplotype and Bz sensitivity in the clinical isolates (14).

### Tissue distribution and drug efflux

The inflammatory response during chronic CD might impact the pharmacokinetics of Bz by influencing drug metabolism enzymes, such as P450, and drug transporter activities (54, 55). Bz also increases its own efflux by upregulating host P-gp and ABC subfamily C member 2 transporter (ABCC2/MRP2) *in vitro* and *in vivo* (56–58). The half-life of Bz is similar after a single dose and multiple dosing over a period of 40 days. However, the maximal Bz concentration in specific organs is significantly higher following a single 100 mg/kg Bz dose compared to that observed in mice administered the same dose daily for 40 days (59, 60).

Bz is rapidly detected in the heart, colon, brain, liver, lungs, and kidneys of chronically infected mice, but maximal concentrations only reached 1.76 ± 0.19 µg/mL in the liver after treatment with a single dose of 100 mg/kg Bz and a total follow-up time of 6 h (59). Depending on the parasite strain, this concentration may be inadequate to kill all *T. cruzi* parasites. IC_50_ concentrations for amastigotes at 72–96 h of treatment are described between 0.64 and 2.18 µg/mL *in vitro* (29). Authors suggest that low absorption during first-pass metabolism in the liver rather than low tissue distribution causes parasitological treatment failure (19). Indeed, no site-specific preference for parasite persistence was observed in a mouse model of acute-stage parasitological treatment failure (25).

Quantification of Bz in human organs is not available. The minimum Bz concentration in human serum under the standard treatment regimen was approximately 6 µg/mL (61), and organ levels may be even lower. Nevertheless, parasitological cure is achievable, indicating that such a concentration may often be sufficient to kill *T. cruzi in vivo*. This may be attributed to the impact of parasite burden and strains and differences in drug susceptibility between *in vitro* assays and the *in vivo* context (62). Furthermore, Bz plasma concentration alone cannot explain treatment success, as the observed Bz plasma concentrations were lower in children than in adults treated with a comparable dose, but the parasitological clearance in children was higher (see below) (63).

### Host drug metabolism

Host drug metabolism may also contribute to inactivating Bz or NFX. Bz in mammals is mainly metabolized in the liver (59). The most abundant phase I metabolite detected through reversed-phase UHPLC–QTOF–MS and UHPLC–QqLIT–MS is Bz-aminoimidazole at about 3% of the administered Bz dose and produced by liver cytochrome P450 (CYP450). Glucuronides of amino-benznidazole were the most abundant detected phase II metabolites (Fig. 2) (43, 59). CYP450-catalyzed metabolism is increased in children compared to adults (64). The weight-corrected clearance rate is higher in children than in adults, possibly contributing to lower Bz steady-state concentrations in children (3.61 mg/L for ages 2–7, 6.88 mg/L for ages 7–12) than in adults (9.69 mg/L) after treatment with 5–8 mg/kg/day Bz. However, even though blood Bz concentrations are lower in children than in adults, *T. cruzi* was killed in all the children that completed the treatment (63, 65), while only 89% of adult patients treated with a comparable dose sustained parasitological clearance 6 months after treatment, as demonstrated by PCR (11). Further investigation of age-dependent Bz metabolism is needed.

Bz metabolites may also be produced in a tissue-specific fashion, contributing to local parasitological treatment failure. For instance, the production of Bz amine differs by over 50-fold between tissues, as measured by HPLC, possibly because of different nitroreduction activities between organs (66).

### Role of the metabolic environment

The metabolic environment differs between tissues, changes after infection, and is impacted by the inflammatory response. *In vitro,* no significant impact of ketoconazole on parasite numbers was observed when added to glutamine-restricted cells due to the reduced production of glutamine-derived 14-methylated sterols, whereas almost all parasites were cleared by 72 h of ketoconazole treatment in the presence of glutamine (67). Lower glutamine peak areas were measured in the large intestine compared to other intestinal sites by metabolomics and might explain parasite survival in certain tissues (68). In parallel, reduced availability of nutrients, such as glucose or glutamine, or fatty acid sources, such as triacylglycerols, impairs the *T. cruzi* proliferation (27, 69, 70). Pre-existing growth impairment from nutrient restriction may increase susceptibility to growth arrest in response to drug treatment (27). These mechanisms would interfere with parasitological cure following treatment with any agent requiring active parasite replication.

## MECHANISMS OF CLINICAL TREATMENT FAILURE

Biomarkers used to monitor parasitological treatment efficacy may not always reflect clinical improvement (71, 72). Instead, clinical readouts focus on cardiac and gastrointestinal symptoms. Mechanisms contributing to clinical treatment failure include irreversible tissue damage and persistent inflammation and metabolic changes (Fig. 1).

### Irreversible tissue damage

Cardiomyocytes are responsible for contraction of the heart essential for blood circulation, but they have a limited capacity for regeneration. When damaged or destroyed by *T. cruzi* infection or immune responses, these cells are replaced by a fibrotic scar tissue. This scar tissue takes the place of the functional muscle, compromising the heart’s ability to contract effectively. The loss of a substantial number of cardiomyocytes diminishes the heart’s pumping efficiency (73–76). Such effects may be prevented by antiparasitic treatment but may not be reversible, although this is challenging to evaluate experimentally since the assessment of cardiac fibrosis in animal models is usually terminal. Alternatively, fibrosis may be reversible when addressed by newer therapeutic regimens (77, 78). Some functional improvement may also be possible, even without reversing fibrosis. For example, treatment with carnitine (see “Inducing metabolic restoration” below) can improve cardiac strain without affecting fibrosis (68). Likewise, resveratrol treatment does not reduce collagen levels but nevertheless significantly improves cardiac function (79).

In parallel, research has shown a marked decrease in parasympathetic nerve cells, especially within the cardiac parasympathetic ganglia, as well as sympathetic denervation following *T. cruzi* infection (80–82). The damage begins during the acute phase of infection when *T. cruzi* causes an inflammatory response leading to neuronal loss, although direct parasite-mediated damage may also be involved (83, 84). Neuronal regeneration is a complex and intricate process that remains incompletely understood but may be at least partially possible (85).

*T. cruzi* and immune cells attack neurons in the enteric nervous system, which disrupts the movement of the colon and the esophagus. This damage results in a loss of coordinated muscle contractions, leading to severe constipation, diarrhea, difficulty swallowing, regurgitation, abdominal pain, and potentially life-threatening dilation of the affected organs (86, 87). Reversibility of these effects is achievable if treatment is administered early in the infection. In a mouse model, successful treatment with Bz at 6 weeks post-infection led to a sustained improvement in gastrointestinal function. However, in animals where parasitological cure was not achieved, the gastrointestinal symptoms of Chagas disease progressively returned. Effective treatment prompted a transition in gene expression from chronic inflammation to tissue repair, marked by increased cell proliferation, elevated expression of glial cell markers, and recovery of neuronal density in the myenteric plexus. Conversely, initiating treatment at 24 weeks post-infection resulted in only partial reversal of symptoms (88).

### Persistent inflammation

Circulating CD4^+^CD8^+^ cell levels improve but do not fully renormalize, even 24–48 months after Bz treatment in both symptomatic and asymptomatic patients (89). Likewise, circulating CD4^+^ activation levels and Treg cell counts also improved in a 12-month follow-up study, although uninfected controls were not included. However, Th17 cells and *T. cruzi-*responsive IFNγ- and TNFα-producing CD4^+^ T cells increased post-treatment (90). While this effect may be temporary (91), it could nevertheless contribute to exacerbating tissue damage. Likewise, high-throughput, real-time qPCR revealed that expression levels in peripheral blood mononuclear cells renormalized 6–12 months post-treatment for only some cytokines and chemokines, such as CCL5 and IL10, whereas *TGFB2* and *IL13* expression levels increased and remained higher than in healthy controls (92). However, a challenge is that these studies cannot confirm parasitological cure; thus, residual parasites may be driving these responses. Nevertheless, these results demonstrate that, even if treatment effectively reduces or clears the parasitic burden, some immune pathways may continue to drive persistent inflammation and tissue injury.

### Persistent metabolic alterations

Metabolomics has played a prominent role in demonstrating that parasitological treatment clearance is insufficient to cause metabolic restoration. Distinct small molecule trajectories were observed in different regions of the heart during chronic infection, with incomplete restoration of these molecules 56 days after treatment with Bz (93). Likewise, even mice that achieved parasitological clearance with Bz still exhibited a urine metabolome similar to those that did not clear parasites 1 month post-treatment (94). In humans, serum metabolomics demonstrated the restoration of some but not all metabolites 3 years after NFX treatment (95). In contrast, similar to what is observed clinically with early patient treatment, for example (96), experimental Bz treatment in acute-stage mouse models successfully restored cardiac metabolism (68).

Given the persistence of these metabolic changes, even after complete parasite clearance (94), and the greater metabolic restoration in mice receiving a combination therapeutic vaccine and low-dose Bz, with higher parasite burden than mice treated with high-dose Bz-only (93), parasite persistence alone is likely insufficient to cause these persistent metabolic changes. In contrast, persistent inflammation, which was not resolved by Bz-only treatment (93), may be maintaining these metabolic patterns, which in turn could be promoting ongoing inflammation.

In parallel, microbiome analyses by 16S sequencing (97, 98) or metagenomics (99, 100) have revealed persistent and worsening disruption of the gut microbiome with *T. cruzi* infection. These effects were restored by Bz treatment in children, who also respond better to Bz treatment (101), whereas adults with and without prior Bz treatment history remained distinct from uninfected controls (99). Given the intersection between intestinal motility and the gut microbiome (102), an altered microbiome composition may be maintained by the irreversible intestinal dysfunction that persists in late-stage CD in mouse models, even after Bz treatment (88). Several bacterial species were correlated with cytokine levels, so, alternatively, microbiome changes may be maintained by ongoing inflammation (100). Differential abundance of microbiome metabolic pathways could be driving the persistent cardiac and urinary metabolome changes observed. For example, specific metabolites persistently decreased in mouse models of infection include purine metabolites (93), and purine synthesis pathway genes were lower in abundance in infected vs uninfected mice (100). Bacterial genes involved in lipid metabolism and amino acid metabolism were likewise decreased in acutely infected mice (100). Determining whether these changes in microbiome genes and gene expression are also observed in the chronic stage of CD is necessary.

Lastly, metabolites directly cause epigenetic changes (103, 104). Differential DNA methylation patterns were observed in CD4^+^ T cells and B cells from the cord blood of newborns from *T. cruzi-*infected mothers but who themselves did not become infected, demonstrating that epigenetic changes can be observed, even without persistent parasite burden (105). One driver may be the elevated urate observed in CD (106), which can cause epigenetic changes affecting immune responses (107). Whether persistent epigenetic changes are observed post-treatment in CD and are a causal determinant of persistent metabolic disruption, even post-treatment remains to be determined.

## ADDRESSING TREATMENT FAILURE

### Higher drug dose to improve parasite clearance

One common way to address unsuccessful parasite clearance is to increase drug dosage (Fig. 1). The bioavailability after a single 100 mg/kg Bz dose in mice is low (59). In contrast, the bioavailability after oral administrations in humans is suggested to be adequate for the killing of *T. cruzi* (108). The recommended treatment dosage based on clinical experience with Bz is 5–8 mg/kg/day for children and 5–10 mg/kg/day for adults (63, 109, 110). Bz treatment doses of 4–6 or 2–3 mg/kg/day, administered for 4 weeks, resulted in negative PCR in 80% of chronic CD patients. Parasites were eliminated faster in the higher-dose group (11). However, safety considerations because of adverse effects like polyneuritis or bone marrow depression preclude dose escalation in human subjects (111).

### Treatment duration

Bz kills *T. cruzi* with a fast kill rate compared to other drugs, such as fexinidazole and posaconazole (50, 112). However, longer treatment duration may help address parasite dormancy. Bz is typically administered over 30 to 60 days (109, 110, 113). Parasitological clearance of trypomastigotes in the blood at the standard Bz dose in chronically infected mice increased from 0% after 20 days to 62% after 40 days (15). To address dormant amastigotes, it has been proposed to administer Bz weekly instead of daily, which allows surviving amastigotes to transform into trypomastigotes or actively replicate amastigotes between drug doses. Unlike dormant amastigotes, trypomastigotes or replicating amastigotes are stages amenable to killing. Treating mice with a 2.5 times higher dose once per week for 30 weeks cleared parasites (114). Dosing twice per week allowed to reduce the treatment period to 8 weeks. Applying a 5-fold higher Bz dose twice per week over a period of 15 weeks successfully killed *T. cruzi* parasites in a primate system after standard treatment strategies failed to clear parasites. However, twice per week treatment for 8 weeks with the Bz standard dose of 100 mg/kg was not effective to eliminate *T. cruzi* in mice that were infected with the *Colombiana* strain for 240 days (115).

In contrast, the BENDITA clinical trial found no additional parasitological cure benefit for an 8-week treatment period compared to 2 or 4 weeks, with parasitological clearance above 80% in all of the treatment groups that received 300 mg/day Bz in patients (11). Treatment-related adverse events occurred more frequently in the longer duration treatment, contributing to non-compliance due to abnormal liver function test results, as well as blood, lymphatic system, skin, and subcutaneous disorders (11). Over half of patients treated with Bz experience adverse effects, leading to treatment discontinuation in 13% of cases (116). Adverse events are more frequent in adults than in children (117). Treatment with NFX is discontinued in almost half of the patients because of adverse events (118). These findings have increased clinician interest in reducing treatment lengths. The Phase 3 NuestroBen Study (NCT 04897516) will investigate 2- and 4-week treatments with 300 mg/day Bz.

### Symptom management

Managing symptoms typically involves medications to address heart failure and arrhythmias, alongside treatments for gastrointestinal problems. For heart-related issues, this may include angiotensin-converting enzyme inhibitors and beta-blockers. In certain cases, patients might require the implantation of pacemakers or defibrillators (76). For managing chronic gastrointestinal symptoms, treatment focuses on symptom relief and quality of life, for example, via dietary adjustments, laxatives, and enemas. In severe cases, surgical options may be necessary (87, 119).

### Inducing metabolic restoration

Considerable tissue metabolic changes are observed in CD (68, 120–122), which have been causally linked to fatal vs non-fatal disease outcomes (68). Thus, improving metabolism could help address clinical treatment failure (Fig. 1). For example, modulating mitochondrial oxidative metabolism improved cardiac function (123). Targeting infection-disrupted pathways, especially those not improved by current antiparasitics, as revealed by LC–MS (93), will be promising. Treatment efficacy in this context would be assessed by metabolomics. Whether a single course of treatment suffices will likely be determined by the mechanisms causing these metabolic alterations (see “Persistent metabolic alterations” above) and whether they are resolved by treatment. Self-maintaining metabolic changes that are not driven by microbiome dysbiosis may be more likely to respond to a single metabolism-modulating course of treatment, for example. Alternatively, one of the proposed metabolism-modulating treatments, L-carnitine (68), may be tolerable for long-term usage. Metabolic restoration, as a treatment approach, is being investigated in other infectious diseases, including influenza virus infection (124), severe acute respiratory syndrome coronavirus 2 infection (125), and sepsis (126, 127). Likewise, trimetazidine, an approved agent for angina in Europe, acts by promoting cardiac glucose oxidation over fatty acid oxidation (128). In parallel, given the intersection between glutamine levels and azole treatment success (67), metabolic modulation may help achieve parasitological cure. Taking advantage of parasite metabolic dependencies could also be effective (129, 130).

### Immunomodulation to improve parasite clearance and reduce disease symptoms

Antiparasitic immune responses are not only critical for *T. cruzi* control but also contribute to tissue damage, providing the impetus for the study of immunomodulation to improve CD symptoms (131). Tested strategies include small molecule drugs, such as pentoxifylline, and neutralizing antibodies, such as anti-TGFβ antibodies, which reshaped cardiac immune responses, reduced cardiac fibrosis, and improved cardiac function in a chronic CD mouse model (132–134), or a therapeutic vaccine (Fig. 1) (135).

### Anti-fibrosis therapies and approaches to promote cardiac regeneration

Fibrosis reversal may be achievable by immunomodulation, although differentiating between reversal of fibrosis vs arresting fibrosis progression is challenging (136). For functional restoration, reversal of fibrosis should be accompanied by cardiomyocyte proliferation. Approaches seeking to restore cardiomyocyte regenerative potential include small molecule agonists of pro-repair pathways, such as Notch signaling, transcription factor over-expression, and stem cell injection (137, 138). Administration of bone marrow-derived mononuclear cells failed to improve CD symptoms in patients, but lack of success could also be due to minimal numbers of stem cells and inability of these cells to differentiate into cardiomyocytes (139). Overall, such approaches await further technical developments outside CD that could lead to reduced costs and greater success rates.

### Combination therapies

Combining drugs with different mechanisms of action may prevent the development of treatment resistance or cure drug-resistant infections. In parallel, combining an antiparasitic with an immunomodulator (“vaccine-linked chemotherapy” [140]) or with a metabolic modulator may be necessary to ensure both parasite clearance and clinical cure. Clinical studies of combination therapies have recently focused on combinations of CYP51 inhibitors with Bz, although their development was halted by results showing an inability to induce parasitological cure in humans (21–23). New anti-*T*. *cruzi* inhibitors are, however, very promising (141). A combination therapy involving reduced dose Bz and a therapeutic vaccine showed superior restoration of cardiac small molecule profiles in a chronic infection mouse model, despite less effective parasite reduction (93).

Amiodarone, an antiarrhythmic drug, was thought to not only manage heart symptoms but also potentially kill *T. cruzi*; however, a subsequent study found amiodarone was ineffective against *T. cruzi in vitro* (142). Bz-amiodarone combination therapy showed enhanced efficacy compared to Bz alone in improving cardiac fibronectin and reactive oxygen species but not the right ventricle area, left ventricle ejection fraction, or stroke volume in chronic mouse models (143). Other studies in mice and humans also suggest limited benefits (142, 144).

Overall, these findings suggest that simultaneously targeting pathogen and host small molecule or immune responses may improve treatment outcomes.

## CHALLENGES AND FUTURE DIRECTIONS

The co-existence of multiple *T. cruzi* stages within an infected cell, with differences in drug sensitivity (20, 28), complicates the anti-*T*. *cruzi* drug development. Understanding *T. cruzi* dormancy and using screening assays that can include killing of dormant amastigotes in rate-of-kill assays will be critical for developing treatments that can ensure parasitological cure with short treatment regimens (112). Single-cell RNA-seq applied to dormant amastigotes would also be extremely beneficial. In addition, slower drug release formulations could help ensure sufficient and persistent drug levels to kill dormant amastigotes (145, 146). Indeed, the role of formulation has been under-appreciated in CD drug development. Sequential parasite whole-genome sequencing and transcriptomics from *in vivo* samples would also clarify parasite evolution during treatment.

Pharmacokinetic studies in CD have been performed at the blood or organ level (19, 54, 59). However, parasite clearance may be heterogeneous between organ regions (147), even between cells (25). The role of the gastrointestinal tract (147) and the influence of the local metabolic environment (67) have been understudied. Furthermore, tissue drug levels do not necessarily imply intracellular drug accumulation. Lower intracellular levels of active drug compounds might be a reason for parasitological treatment failure, as demonstrated in cancer chemotherapy by quantitative single cell mass spectrometry (148). Identification and quantification of drug metabolites across cells and parasite life stages are currently lacking. A comprehensive spatial approach to pharmacokinetics and CD drug development that includes single-cell measurements is thus necessary.

From a patient perspective, clinical treatment success is the goal: return to health with disappearance of disease symptoms. However, due to the slow progression of CD, recent clinical trials have understandably been designed with parasitological rather than clinical efficacy readouts (11, 22). One exception is the BENEFIT clinical trial, which provided the critical insight that parasite clearance in a symptomatic patient population is insufficient to prevent cardiac events or mortality, demonstrating, at least in that patient group, a disconnect between parasitological and clinical treatment success (10). Faster readouts of clinical treatment success are strongly needed so that clinical trials can incorporate both clinical and parasitological readouts in their design. Metabolic indicators or alternative echocardiographic measurements are showing promise as indicators of disease progression and could serve to predict the clinical outcome (93, 149), while combinations of new immune biomarkers may represent better readouts of parasitological cure (150–152). Given the complex and multifactorial nature of Chagas disease, omics analyses will play a prominent role in identifying and validating these biomarkers.

Likewise, given the BENEFIT clinical trial’s findings (10), there is a need for increased inclusion of drug development strategies that focus on improving symptoms, including via immune modulation or metabolic modulation, to ensure clinical cure. Such new treatments should include system-scale readouts of efficacy. Most efforts have focused on cardiac CD, but new mouse models of gastrointestinal CD will help with the development of interventions to address gastrointestinal symptoms (153).

Overall, continued investment in both fundamental and translational CD research will be critical to address these gaps and deliver treatments that can ensure clinical cure.
